# A novel cuproptosis-related gene signature to predict prognosis in Glioma

**DOI:** 10.1186/s12885-023-10714-8

**Published:** 2023-03-13

**Authors:** Mengyang Zhang, Xiaobai Liu, Di Wang, Xuelei Ruan, Ping Wang, Libo Liu, Yixue Xue

**Affiliations:** 1 Department of Neurobiology, School of Life Sciences, China Medical University, Shenyang, 110122 China; 2 Key Laboratory of Neuro-Oncology in Liaoning Province, Shenyang, 110004 China; 3 Department of Neurosurgery, Shengjing Hospital of China Medical University, Shenyang, 110004 China

**Keywords:** Glioma, Cuproptosis, Prognostic model, Proliferation

## Abstract

Glioma is primary brain tumour with a poor prognosis. Metabolic reprogramming is a hallmark of glioma, and is critical in the development of antiglioma agents and glioma therapy. Cuproptosis is a novel form of cell death mediated by protein lipidation and highly associated with mitochondrial metabolism. However, the clinical impact of cuproptosis-related genes (CRGs) in glioma remains largely unknown. The purpose of this study is to create a new CRGs signature that can be used to predict survival and immunotherapy in glioma patients. LASSO regression analysis was applied to establish prognostic gene signatures. Furthermore, a CRGs signature-based nomogram was developed and demonstrated good predictive potential. We also analyzed the relationship of CRGs and immune infiltration and the correlation with the pathological grade of glioma. Finally, we explored the miRNA that may regulate cuproptosis-related gene FDX1. We found that miR-606 was markedly downregulated in GBM, overexpression of miR-606 can significantly inhibit aerobic glycolysis and proliferation of GBM cells. FDX1 was upregulated in GBM, knockdown of FDX1 significantly inhibit aerobic glycolysis and proliferation of GBM cells. And luciferase assay was used to verified that miR-606 binds to and regulates FDX1 mRNA. These results provide a basis for further exploring the biological mechanisms of cuproptosis. This study may provide new potential therapeutic perspectives for patients with glioma.

**Supplementary Information:**

The online version contains supplementary material available at 10.1186/s12885-023-10714-8.

## Introduction

Glioma is the most common primary tumor in the brain, accounting for 81% of the central nervous system (CNS), malignant tumors [1]. According to the World Health Organization (WHO) classification, gliomas are classified into 4 grades, with grade I and II gliomas indicating low grade, and grade III and IV gliomas indicating high grade gliomas (HGG) [2]. Often, relatively higher grades are associated with a poorer prognosis. The 10-year survival rate for low-grade gliomas was 47%, with a median survival time of 11.6 years [3]. For HGG, the median overall survival (OS) in patients with grade III glioma is approximately 3 years, while the median OS time in grade 4 glioma was poor at 15 months [4]. However, the complex biological properties of glioma cells, such as highly heterogeneity, highly diffuse ability, immunosuppressive tumor microenvironment and development of drug resistance, often limiting the efficacy of existing therapies [5, 6]. Therefore, it is urgent to find potential biomarkers and develop more effective prognostic models, so as to improve the glioma patient prognosis.

The concept of copper-dependent cell death, termed cuproptosis, was first proposed in study published in *Science* by Tsvetkov et al [7]. The researchers found a new mechanism different from the known cell death: a copper-dependent and regulated mode of cell death, through the direct binding of copper to the lipoylated components of tricarboxylic acid (TCA) cycle, leading to the abnormal aggregation of lipoylated proteins and the loss of iron-sulfur tusters, thus leading to proteotoxic stress responses ultimately leading to cell death [8]. Copper is an indispensable trace element involved in various biological processes. Recent studies have shown significantly elevated copper levels in serum and tumor tissues in cancer patients compared with healthy patients [9–11]. And copper is associated with tumorigenesis, invasion, and metastasis [12–15].

However, there are no reports on the effect of cuproptosis regulatory mechanisms on glioma. Therefore, in this study, we explored the possible prognostic value of cuproptosis-related genes (CRGs) in glioma, and explored the regulatory mechanism of upstream factors on CRGs.

## Materials and methods

### Clinical specimens

This study was approved by the Institutional Review Board of the Shengjing Hospital of China Medical University. Human glioma and normal brain specimens and were obtained from the Department of Neurosurgery of the Shengjing Hospital, China Medical University. All participants signed and provided informed consent. Detailed patient information of 9 samples was shown in Supplementary Table 1.

### Data source and preprocessing

The RNAseq data in the TPM format for TCGA and GTEx, as uniformly processed by the Toil process in UCSC XENA (https://xenabrowser.net/datapages/)[16]. The corresponding normal tissue data from the GBMLGG (glioma) and GTEx of TCGA were extracted. RNAseq data in TPM (transcripts per million reads) format and log2 transformation. RNA-seq data of 689 samples derived from TCGA and 1157 samples derived from GTEx were included.

### Correlation analysis

RNAseq data in level 3 HTSeq-FPKM format in the TCGA GBMLGG (glioma) project was converted to TPM (transcripts per million reads) format and log2 transformed. Analysis of correlation was performed using the Pearson correlation test.

### GO | KEGG enrichment analysis

Software R was used for statistical analysis and visualization (version 3.6.3). ggplot2 package was used for visualizations (version 3.3.3); clusterProfiler package was used for analysis of selected data (version 3.14.3). This analysis was performed to retrieve the corresponding Entrez IDs of the molecules in the input list followed by hypergeometric analysis using the GO KEGG library and obtain analysis results. The significance cut-off value for GO | KEGG enrichment analysis was set to a corrected *p*-value < 0.05.

### Immune infiltration analysis

Software R was used for statistical analysis and visualization (version 3.6.3). R package: GSVA package (version 1.34.0) [17]. Immune infiltration algorithm: ssGSEA (GSVA package built-in algorithm). Data: The RNAseq data in the level 3 HTSeq-FPKM format in the TCGA GBMLGG (glioma) project. The RNAseq data in the FPKM format was converted to the TPM format and was log2-transformed. Immunocyte: aDC [activated DC]; B cells; CD8 T cells; Cytotoxic cells; DC; Eosinophils; iDC [immature DC]; Macrophages; Mast cells; Neutrophils; NK CD56bright cells; NK CD56dim cells; NK cells; pDC [Plasmacytoid DC]; T cells; T helper cells; Tcm [T central memory]; Tem [T effector memory]; Tfh [T follicular helper]; Tgd [T gamma delta]; Th1 cells; Th17 cells; Th2 cells; Treg. The markers of the 24 immune cells came from an Immunity article, which were specifically classified and described in this literature [18].

### Clinical relevance

Software R was used for statistical analysis and visualization (version 3.6.3). ggplot2 package was used for visualizations (version 3.3.3). WHO grade Data was used as clinical variable. RNAseq data and clinical data in the level 3 HTSeq-FPKM format in the TCGA GBMLGG (glioma) project was converted to TPM format and log2 transformed. Three data of WHO grade, IDH mutation status, and 1p / 19q deletion were obtained from the study of Ceccarelli et al [19].

### Kaplan–Meier curve

Software: R (version 3.6.3) (statistical analysis and visualization) R package: survminer package was used for visualization (version 0.4.9), survival package was used for statistical analysis of survival data (version 3.2–10). RNAseq data and clinical data in the level 3 HTSeq-FPKM format in the TCGA GBMLGG (glioma) project was converted to TPM format and log2 transformed. Prognosis type: Overall Survival. Prognostic data were obtained from an article by Cell [20].

### Real-Time PCR (qRT-PCR) assay

Total RNA was extracted from tissues (NBT, LGG, and HGG) and cells (U251 and U373) with Trizol reagent (Life Technologies Corporation, Carlsbad, CA, USA). One-Step SYBR PrimeScript primary RT-PCR kit (Takara Bio, Beijing, China) was used to evaluate the expression levels of *FDX1* mRNA, and the reaction was carried out by the 7500 Fast RT-PCR system. β-actin was used as the endogenous control. Reverse transcription of miR-606 was performed using TaqMan MicroRNA Reverse Transcription kit (Applied Biosystems, Foster City, CA, USA); then the expression of miR-606 were detected with TaqMan Universal Master Mix II. U6 was used as the endogenous control. The primers are shown in Supplementary Table 2.

### Western blot assay

For western blot assay, in vitro lysed cell protein was prepared. FDX1 antibody (12,592–1-AP; 1:1000 dilution; Proteintech) and β-actin antibody (66,009–1-Ig; 1:10,000 dilution; Proteintech) were used for western blot assay according to the manufacturer’s protocol. Full length uncropped original western blots used in the manuscript are shown in Supplementary Information.

### Luciferase reporter assay

The binding sites of miR-606 and FDX1 predicted by IntaRNA database were used in luciferase reporter assay. Wild-type (Wt) and mutant (Mut) FDX1 3'UTR luciferase reporter recombinant plasmid were constructed and transfected into HEK-293 T cells. The relative luciferase activity was measured according to the kit instructions.

### Measurement of extracellular acidification rate

The ECAR of the cells was measured with XF-24 extracelluar flux analyzer (Seahorse Biosciences) following standard operating procedures. Cells were seeded into Seahorse XF24 V7 PS Cell Culture Microplates (5 × 10^4^ per well) and cultured overnight. ECAR was measured in XF base medium supplemented with 2 mM glutamine (pH = 7.4). Subsequently, glucose (10 mM), oligomycin (1 μM), and 2-DG (50 mM) were added in XF base medium to continue the measurement and analysis.

### Lactate production and glucose utilization measurementassays

The lactate concentration in the culture medium was measured by using a lactate test kit (Jiancheng, China). The glucose concentration in the culture medium was measured by using a glucose measurement kit (Jiancheng, China).

### Cell proliferation assay

Cell proliferation was evaluated by Cell Counting Kit-8 (CCK-8, Beyotime Institute of Biotechnology, China). Cells were seeded in 96-well plates at the density of 2000 cells per well. After 72 h, 10 μl CCK-8 solution was added and cells were incubated at 37 °C for 2 h. The absorbance was measured at 450 nm on the SpectraMax M5 microplate reader (Molecular Devices, USA).

### Statistical analysis

The experimental data were expressed as the mean ± standard deviation (SD) and were analyzed using GraphPad Prism v8.4 statistical software. Differences between groups were analyzed using a Student’s t-test or one-way ANOVA. Software R was used for statistical analysis and visualization (version 3.6.3). R Package: ggplot2 package was used for visualizations (version 3.3.3). Significance identification: ns, *p* >  = 0.05; *, *p* < 0.05; **, *p* < 0.01; ***, *p* < 0.001.

## Results

### Expression and functional enrichment analysis of CRGs in Glioma

**Fig. 1:**
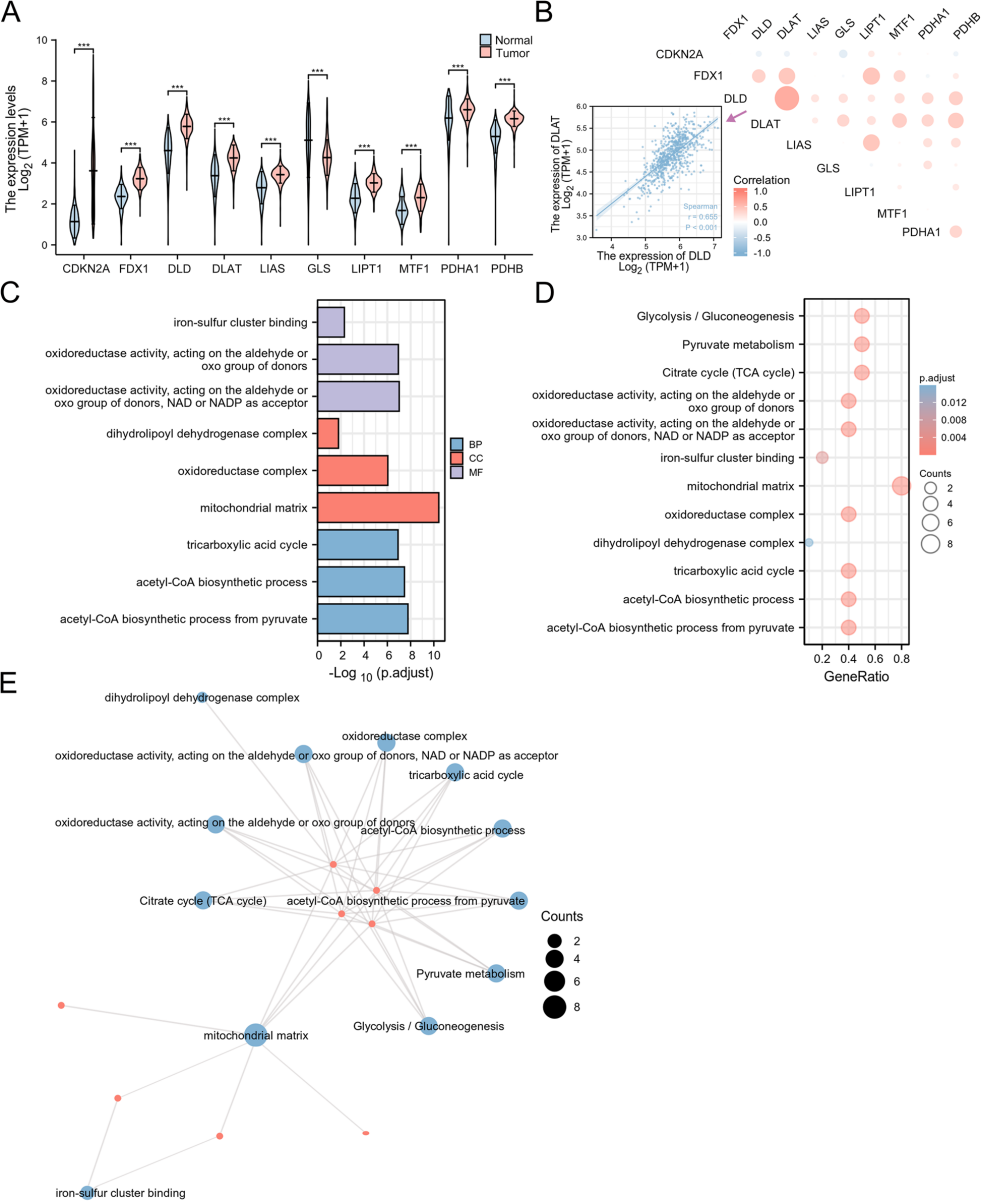
Expression and functional enrichment analysis of CRGs in Glioma. **A** The expression of 10 CRGs in glioma and normal tissues (tumor in red and normal in blue). CRG: cuproptosis-related gene. **B** Correlations between the expression of cuproptosis regulators. **C**, **D** The enriched item in the gene ontology (GO) analysis and Kyoto Encyclopedia of Genes and Genomes (KEGG) analysis [21–23]. **E** Functional enrichment analysis visualization network. The size of circles represents the number of enriched genes. BP: biological process, CC: cellular component, and MF: molecular function

We collated 10 genes closely related to the function of cuproptosis from a study published in *Science* by Tsvetkov et al. (CDKN2A, FDX1, DLD, DLAT, LIAS, GLS, LIPT1, MTF1, PDHA1 and PDHB) [7]. Figure 1A showed that CDKN2A, FDX1, DLD, DLAT, LIAS, LIPT1, MTF1, PDHA1, and PDHB were highly expressed in the tumors of TCGA glioma patients than in normal tissues, and the GLS expression was significantly reduced. In addition, we found a correlation between CRGs by correlation analysis (Fig. 1B). We further analyzed the biological function of CRGs by using the GO and KEGG databases. Figure 1C showed the biological processes associated with CRGs. KEGG pathway enrichment analysis showed that these CRGs were related to the Citrate cycle (TCA cycle), pyruvate metabolism and glycolysis/gluconeogenesis (Fig. 1D). Figure 1E showed the functional enrichment analysis visualization network.

### Construction of the prognostic signature of CRGs in Glioma

**Fig. 2:**
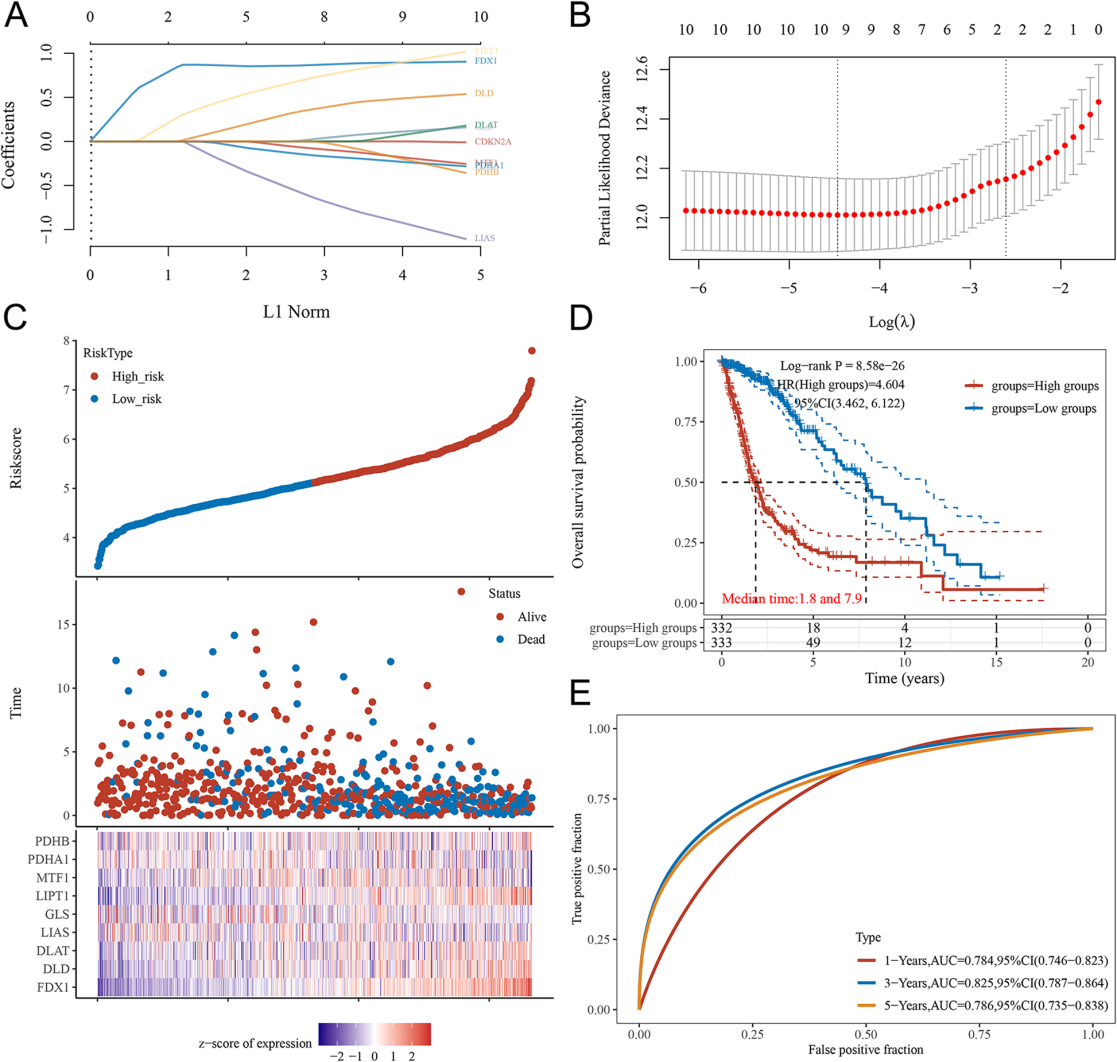
Construction of the prognostic signature of CRGs in Glioma.** A** LASSO coefficient profiles of CRGs in glioma. A coefficient profile plot was generated against the log (lambda) sequence. **B** Selection of the optimal parameter (lambda) in the LASSO model for glioma. **C** Distribution of risk score, survival status and the expression of prognostic CRGs. **D** Kaplan–Meier survival analysis of the CRGs signature from TCGA dataset, comparison among different groups was made by log-rank test. **E** The ROC curve of CRGs. The higher values of AUC corresponding to higher predictive power

Further analysis of the relationship between CRGs and the prognosis of gliomas revealed that high expression of FDX1, DLD, DLAT, and LIPT1 was associated with poorer survival in glioma patients, and high expression of CDKN2A and PDHA1 was significantly associated with better survival in gliomas. Further, we established prognostic gene signatures using the 10 CRGs via LASSO regression analysis (downscaling and building of prognostic models; the number of genes included in the model will not necessarily equal the number of input genes) (Fig. 2A, B). For OS outcomes of glioma patients, a 9-gene signature was selected to construct the prognostic score using their regression coefficients: Riskscore = (0.8949)*FDX1 + (0.4902)*DLD + (0.0778)*DLAT + (-0.9324)*LIAS + (0.1132)*GLS + (0.903)*LIPT1 + (-0.1847)*MTF1 + (-0.2352)*PDHA1 + (-0.2045)*PDHB. The relationships of risk scores, survival time and survival status for the selected datasets are shown in Fig. 2C. Kaplan–Meier survival analysis showed patients with high risk scores had significantly shorter survival times than those with low risk scores, the median times were 1.8 years and 7.9 years, respectively (Fig. 2D). We used ROC curves to assess the predictive value of the OS risk score (Fig. 2E). The prediction accuracy for the 1-year, 3-year and 5-year ROC curves was 0.784, 0.825 and 0.786, respectively. The above results indicated that the cuproptosis-related risk signature we constructed showed a significant association with survival of glioma.

### Nomogram development and validation for Glioma

**Fig. 3:**
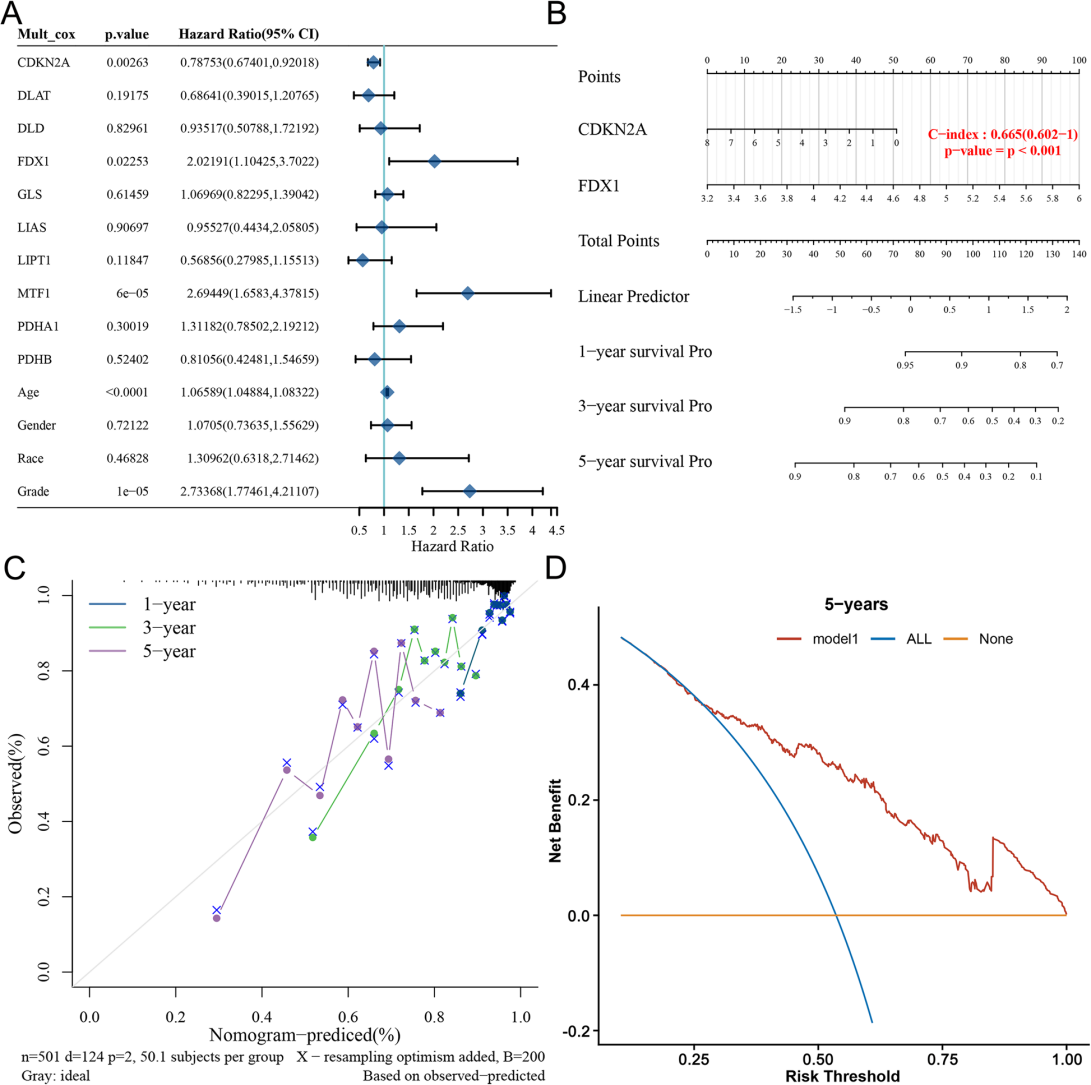
Nomogram development and validation for Glioma. **A** Multivariate Cox regression considering clinical information and prognostic CRGs in glioma (OS). **B** Nomogram to predict the 1-year, 3-year and 5-year OS rate of glioma patients.** C** Calibration curve for the OS nomogram model in glioma. dashed diagonal line represents the ideal nomogram, and the blue line, red line and orange line represent the 1-year, 3-year and 5-year of the observed nomogram. **D** Decision curve analysis. The x-axis was determined by the threshold probability, at which the harm of false-positive intervention exceeded the harm of a false-negative non-intervention, and thus an intervention was triggered. The y-axis was a net benefit, which was the relative benefit derived from the proportion of true-positive results subtracted from the proportion of false-positive results weighted by a ratio of threshold probabilities. Under the same probability, the clinical usefulness was better when the net benefit was higher

We further integrated clinical information and genetic characteristics of TCGA patients to develop the nomogram performed the multivariable Cox regression model (Fig. 3A-C). The c-index (0.665) for OS reflects the good predictive performance of the nomogram (Fig. 3B). The calibration plots showed favorable concordance between predicted and observed OS at survival at years 1, 3 and 5 (Fig. 3C). Meanwhile, decision curve analysis was used to evaluate the clinical utility of the CRGs score (Fig. 3D). None and ALL are two reference lines, and the curve of the other models being closer to the two reference lines indicate less usefulness; the higher the ordinate, the better the model. If the ordinate in the larger (horizontal coordinate) threshold interval is higher than the value of other models, the model is better.

### Correlation between expression of CRGs and immune infiltration levels in Glioma

**Fig. 4:**
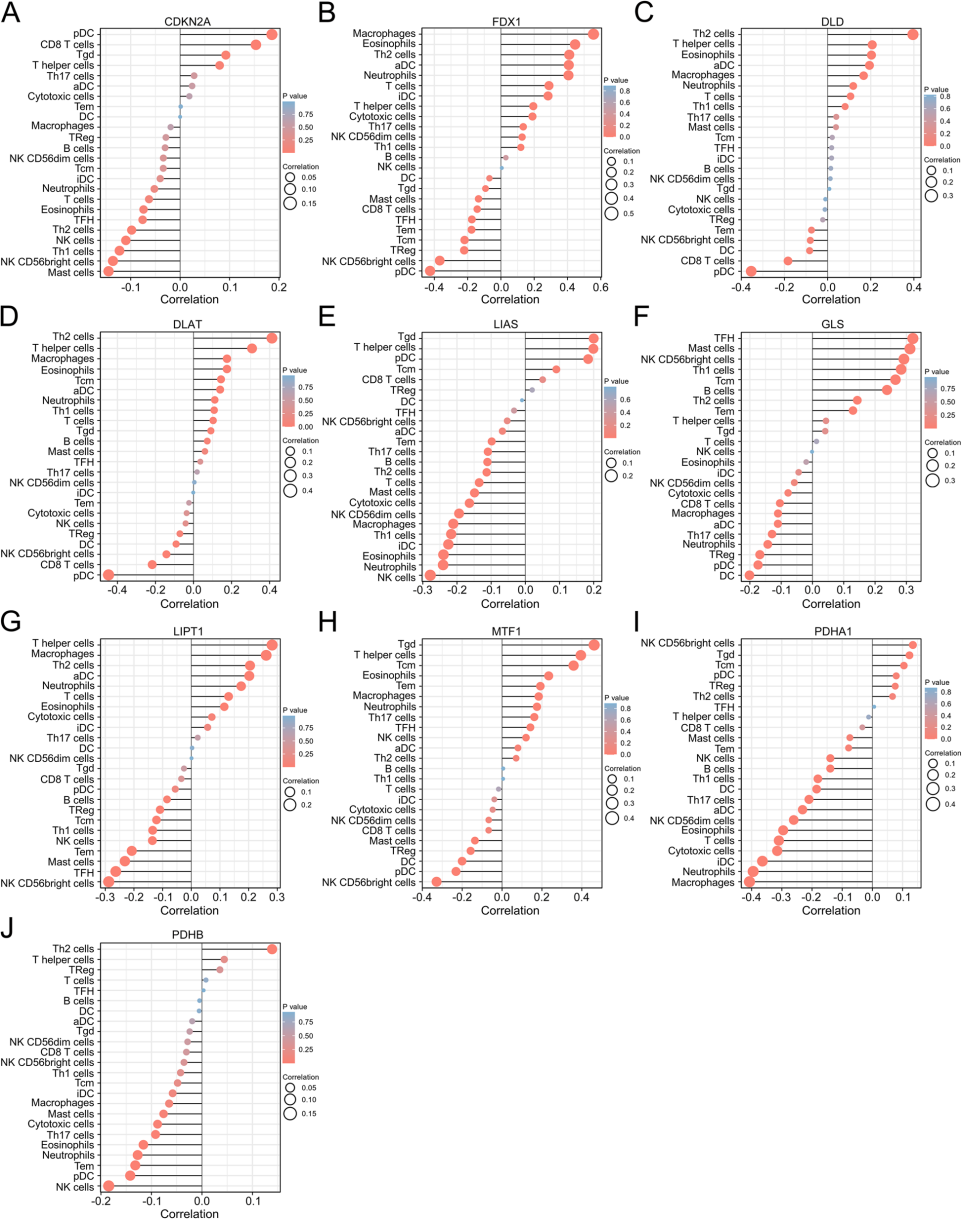
Correlation between expression of CRGs and immune infiltration levels in Glioma. **A-J** The size of the circle represents the degree of correlation, with a higher correlation indicated by a relatively larger circle. The higher the height of the rod (the distance from 0), the higher the correlation degree (a positive number represents a positive correlation, and a negative number represents a negative correlation). The circle color represents the *P* value obtained via the statistical method to determine the correlation. In this study, a Pearson correlation coefficient greater than 0.3 and with *P* < 0.05 was considered a positive correlation, and a Pearson correlation coefficient less than − 0.3 with *P* < 0.05 was considered a negative correlation

We next analyzed the relationship between CRGs and immune infiltration in glioma to assess whether CRGs could affect the immune cell recruitment in the tumor microenvironment and hence the glioma prognosis. The expression level of FDX1 was positively associated with the immune infiltration level of neutrophils, aDC, Th2 cells, eosinophils, and macrophages and negatively correlated with pDC and NK CD56bright cells. The expression level of DLD was positively associated with the immune infiltration level of Th2 cells and negatively correlated with pDC. The expression level of DLAT was positively associated with the immune infiltration level of Th2 and T helper cells while negatively correlated with pDC. The expression level of GLS was positively associated with the immune infiltration level of TFH, mast cells and NK CD56bright cells. The expression level of LIPT1 was positively associated with the immune infiltration level of T helper cells and negatively correlated with NK CD56bright cells. The expression level of MTF1 was positively associated with the immune infiltration level of Tgd, Tcm and T helper cells. The expression level of PDHA1 was negatively associated with the immune infiltration level of T cells, cytotoxic cells, iDC, neutrophils and macrophages (Fig. 4A-J).

### Correlation between FDX1 expression and 8 immune checkpoints

**Fig. 5:**
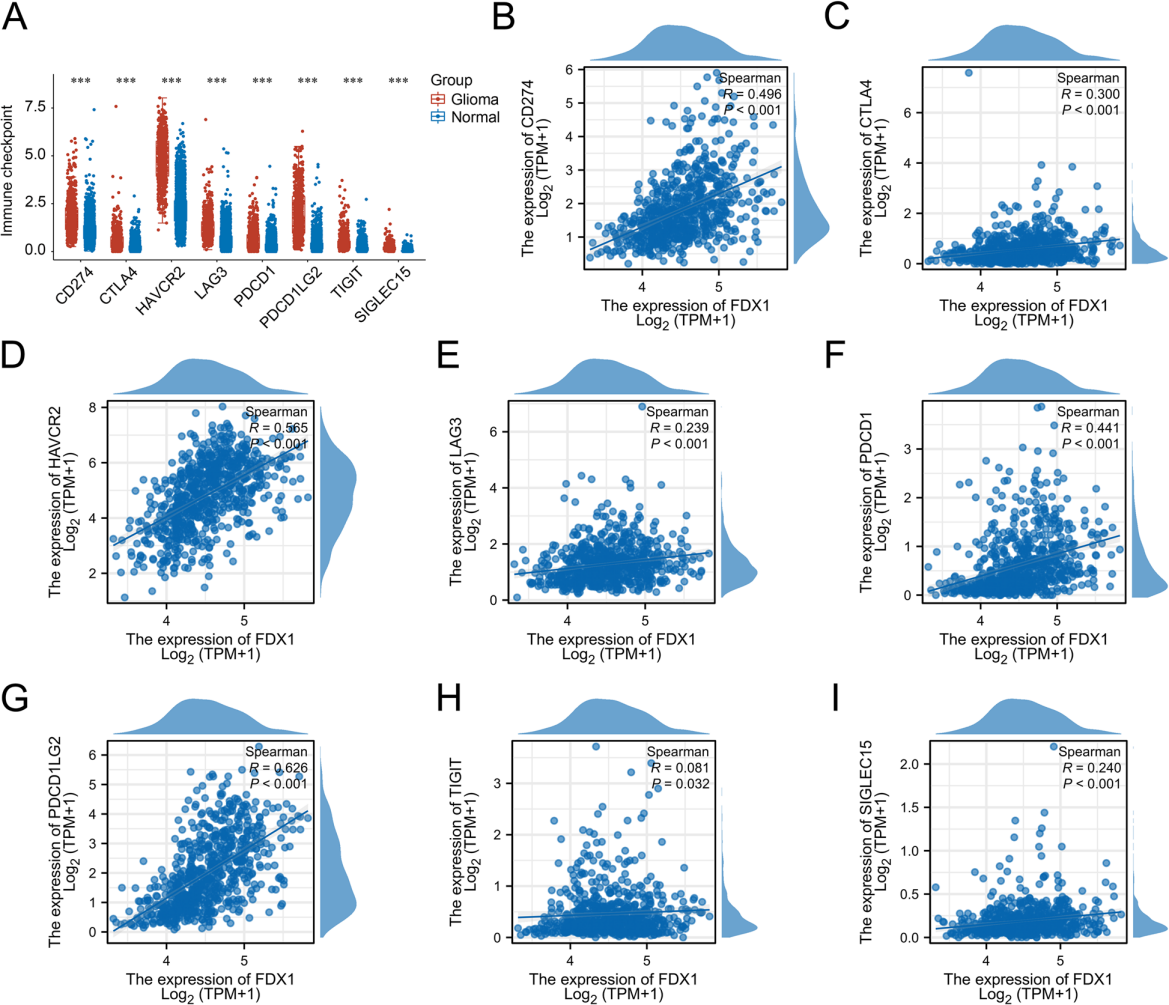
Correlation between FDX1 expression and 8 immune checkpoints. **A** The expression distribution of immune checkpoints gene in glioma tissues and normal brain tissues. Statistical differences between the two groups were compared using the Wilcox test, *p < 0.05, **p < 0.01,***p < 0.001. **B**-**I** Correlation between FDX1 expression and 8 immune checkpoints

We further analyzed the expression distribution of immune checkpoints gene (including CD274, CTLA4, HAVCR2, LAG3, PDCD1, PDCD1LG2, TIGIT, and SIGLEC15) in glioma tissues and normal brain tissues using the TCGA database (Fig. 5A). Then we analyzed the association between FDX1 expression and 8 immune checkpoints. The results showed a significant positive correlation between FDX1 expression and the expression of CD274, HAVCR2, PDCD1, and PDCD1LG2 (*R* > 0.3 and *P* < 0.001) (Fig. 5B-I).

### Differential expression of CRGs in Glioma of different grades

**Fig. 6:**
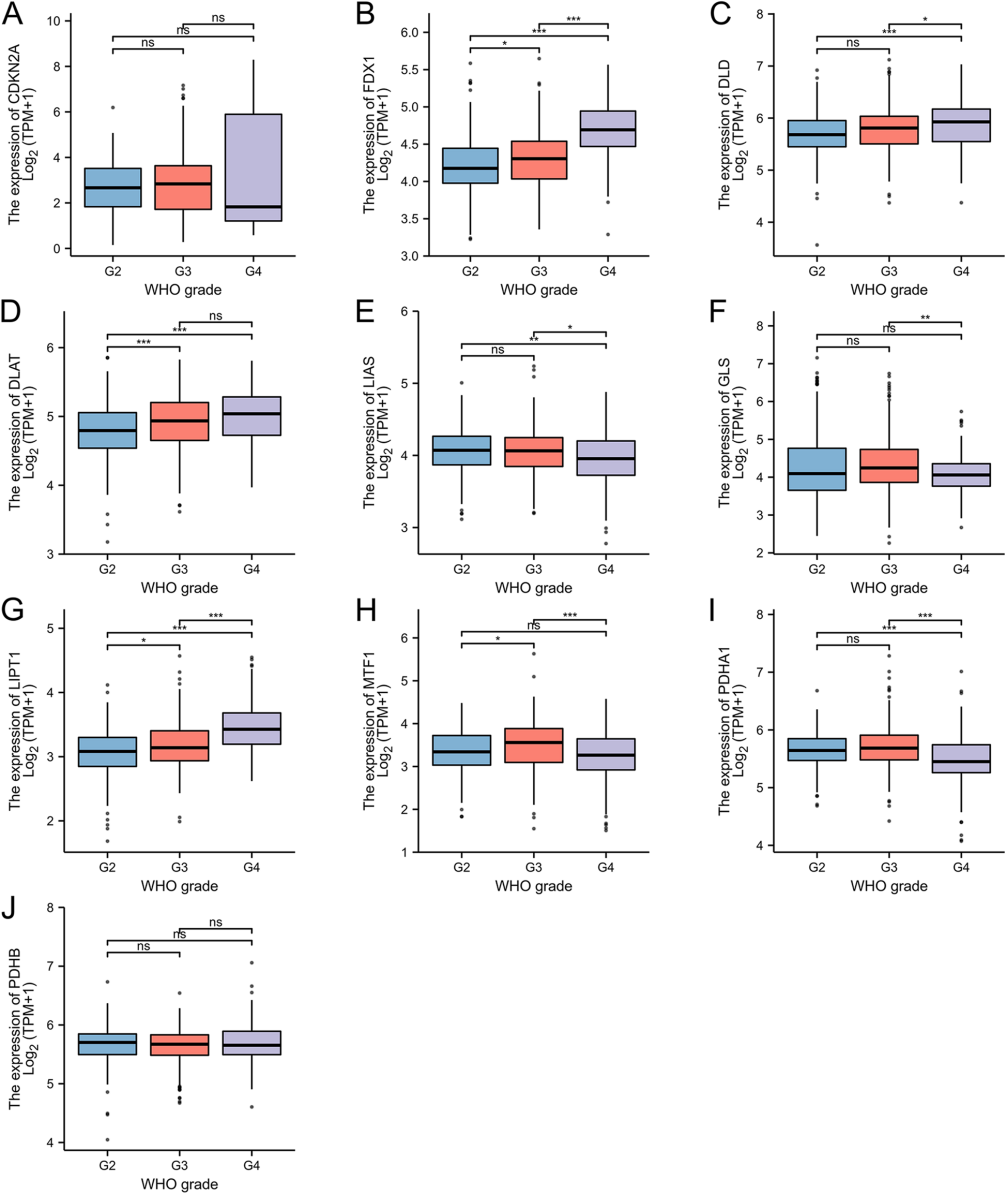
Differential expression of CRGs in Glioma of different grades. **A**-**J** Differential expression of CRGs in glioma of different grades

The expression levels of FDX1 and LIPT1 varied in different histological grades of glioma, as the glioma grade was increased, there was an upward trend of FDX1 and LIPT1 expression. DLD and PDHA1 were showed significantly higher expression in G4 than G2. While CDKN2A and PDHB did not show any statistical difference across histological grades. These results suggested that the expression of CRGs may be correlated with disease grade and the presence of necrosis of glioma (Fig. 6A-J).

### Interactions forecasting of Differential Expression MicroRNAs (DEmiRNAs) with CRGs in glioma tissues

**Fig. 7:**
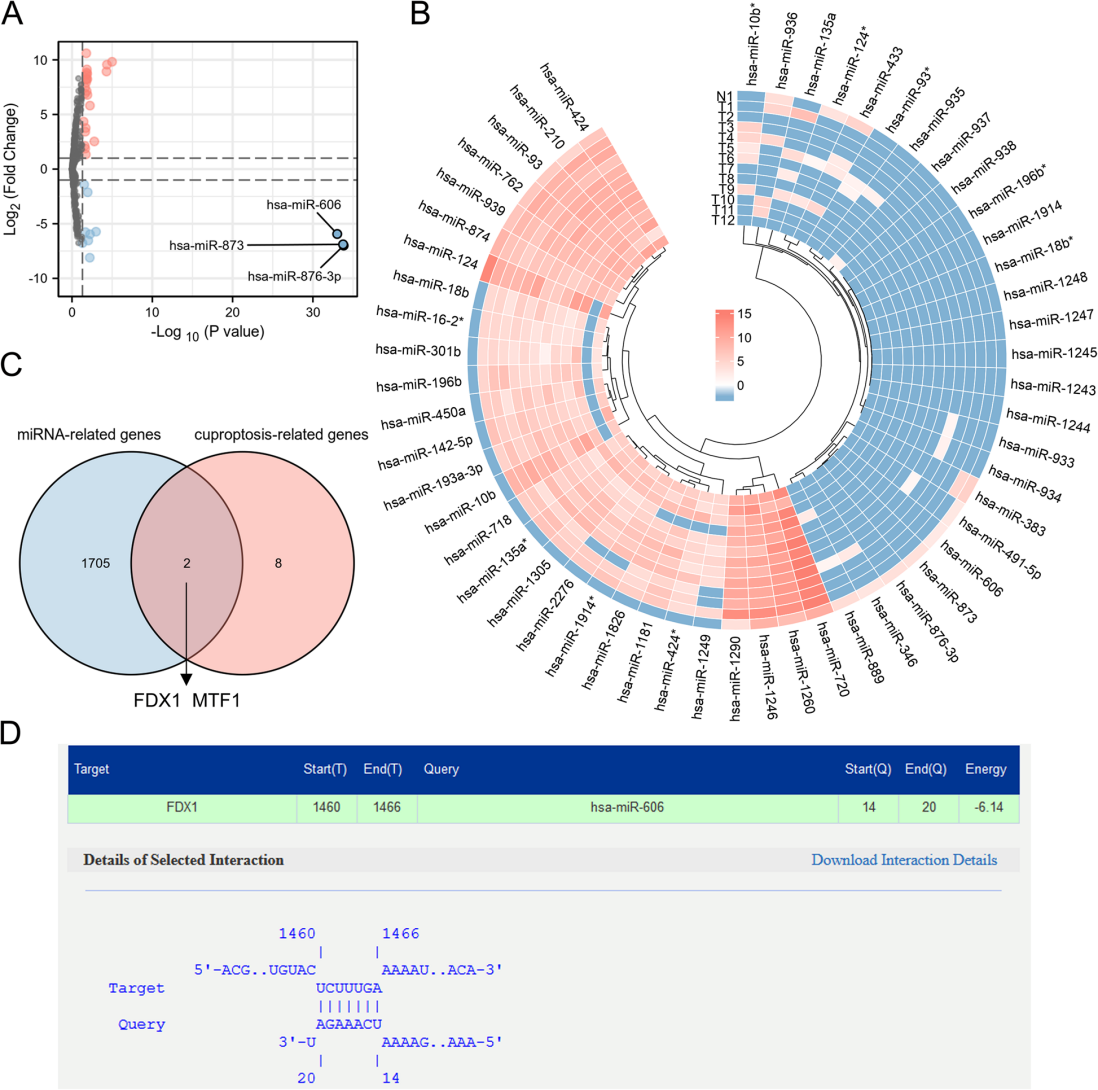
Interactions forecasting of DEmiRNAs with CRGs in glioma tissues. **A**, **B** Volcano map and heat map showed DEmiRNAs in glioma tissues compared to normal human brain. **C** Venn graph showing the 2 overlapped target genes identified with intersection of two gene sets. **D** Bnding sites of FDX1 with hsa-miR-606 analyzed using IntaRNA database

To further analyze whether there are upstream factors to regulate CRGs, miRNA microarrays datasets GSE61710 was used to analyze DEmiRNAs in normal brain tissue versus glioma tissue, the results show that there were 22 up-regulated and 10 down-regulated miRNAs (|log2(FC)|> 2 & p-value < 0.05) (Fig. 7A, B). Then we entered the above DEmiRNAs list for miRNA-mRNA interactions forecasting at mirDIP v4.1 (http://ophid.utoronto.ca/mirDIP/), and took the intersection of the predicted target mRNA with the CRGs, Fig. 7C showed two target mRNAs we obtained (*FDX1* mRNA binds to hsa-miR-606; *MTF1* mRNA binds to hsa-miR-193a-3p). We note that, FDX1 showed a strong positive correlation with the poor prognosis and pathological grade of glioma in the above results, however, MTF1 did not show a significant association with the poor prognosis or the pathological grade of glioma. So we further analyzed the binding site of FDX1 with miR-606, using IntaRNA database (http://rna.informatik.uni-freiburg.de/IntaRNA) (Fig. 7D).

### MiR-606 targets 3’UTR of FDX1 mRNA

**Fig. 8:**
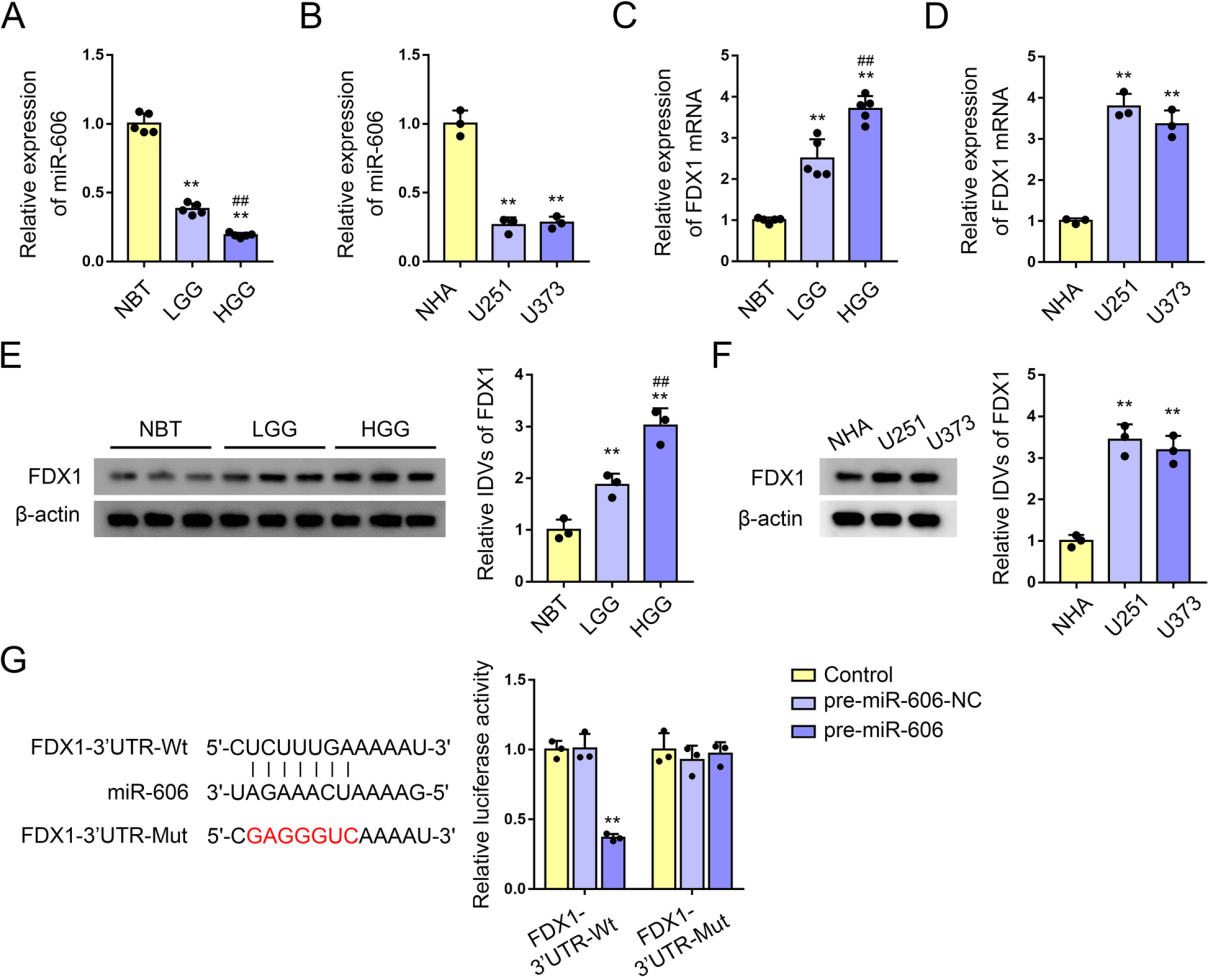
MiR-606 targets 3’UTR of FDX1 mRNA.** A** qRT-PCR was used to detect the expression of miR-606 in NBT, LGG and HGG. Each value represents the mean ± SD (*n* = 5, each group), **P < 0.01, compared with NBT group; ^##^*P* < 0.01, compared with LGG group. **B** qRT-PCR was used to detect the miR-606 expression level in NHA, U251 and U373 cells. Each value represents the mean ± SD (*n* = 3, each group), **P < 0.01, compared with NHA group. **C** qRT-PCR was used to detect the mRNA expression of FDX1 in NBT, LGG and HGG. Each value represents the mean ± SD (*n* = 5, each group), **P < 0.01, compared with NBT group; ^##^*P* < 0.01, compared with LGG group. **D** qRT-PCR was used to detect the FDX1 mRNA expression level in NHA, U251 and U373 cells. Each value represents the mean ± SD (*n* = 3, each group), **P < 0.01, compared with NHA group. **E** Western blot was used to detect the expression of FDX1 in NBT, LGG and HGG. Each value represents the mean ± SD (*n* = 3, each group), **P < 0.01, compared with NBT group; ^##^*P* < 0.01, compared with LGG group. **F** Western blot was used to detect the expression of FDX1 in NHA, U251 and U373. Each value represents the mean ± SD (*n* = 3, each group), **P < 0.01, compared with NHA group. **G** The predicted miR-606 binding sites in FDX1-3’UTR-Wt and the designed mutant sequence. Relative luciferase activity in HEK293T cells co-transfected with FDX1-3’UTR-Wt or FDX1-3’UTR-Mut and pre-miR-606 or pre-miR-606-NC. Data represent means ± SD (*n* = 3, each group). **p < 0.01 versus the FDX1-3’UTR-Wt + pre-miR-606-NC group

We performed qRT-PCR in normal brain tissues (NBT), low-grade glioma (LGG, WHO grades I-II) and high-grade glioma (HGG, WHO grades III-IV), results showed that miR-606 expression levels were negatively correlated with histopathological grading (Fig. 8A). Then we performed qRT-PCR in two GBM cell lines (U251 and U373 cells) and found that miR-606 expression was significantly decreased in U251 and U373 cells (Fig. 8B). Then qRT-PCR and western blotting results showed that FDX1 expression levels were positively correlated with histopathological grading (Fig. 8C, E), and significantly upregulated in U251 and U373 cells (Fig. 8D, F). Then luciferase assay showed that the luciferase activity was significantly reduced in the FDX1-3’UTR-Wt (FDX1 wild-type) + pre-miR-606 group compared with the FDX1-3’UTR-Wt + pre-NC group. However, luciferase activity in the the FDX1-3’UTR-Mut (FDX1 mRNA and miR-606 binding sites mutant) + pre-miR-606 group were not statistically significant compared with the the FDX1-3’UTR-Mut + pre-NC group (Fig. 8G).

### Overexpression of miR-606 inhibits the glycolysis and proliferation of GBM cells; knockdown of FDX1 inhibits the glycolysis and proliferation of GBM cells

**Fig. 9:**
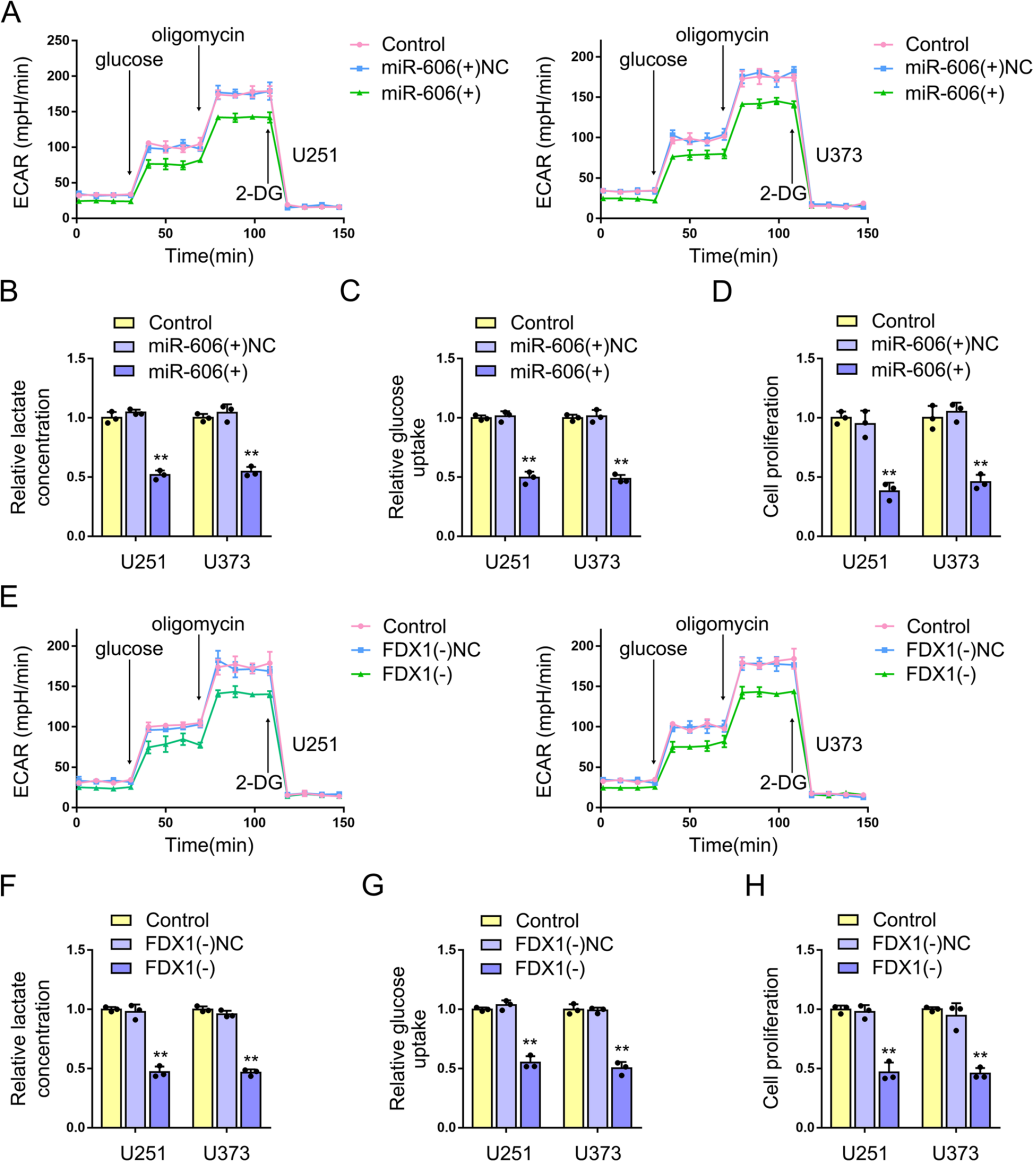
Overexpression of miR-606 inhibits the glycolysis and proliferation of GBM cells; knockdown of FDX1 inhibits the glycolysis and proliferation of GBM cells. **A** ECAR assays were used to detect the effect of miR-606 overexpression on the extracellular acidification rate in U251 and U373 cells. Data represent means ± SD (*n* = 3, each group). **B-D** The effect of miR-606 overexpression on lactate production, glucose consumption and cell proliferation were measured in U251 and U373 cells. Data represent means ± SD (*n* = 3, each group), **P < 0.01, compared with miR-606( +)NC group. **E** The effect of knockdown FDX1 on ECAR in U251 and U373 cells. Data represent means ± SD (*n* = 3, each group). **F–H** The effect of knockdown of FDX1 on lactate production, glucose consumption and cell proliferation were measured in U251 and U373 cells. Data represent means ± SD (*n* = 3, each group), **P < 0.01, compared with FDX1(-)NC group

We next overexpressed miR-606 in U251 and U373 cells to examine changes in cellular aerobic glycolysis levels and proliferation capacity. Extracellular acidification rate (ECAR) experiments showed that overexpression of miR-606 significantly reduced aerobic glycolysis in U251 and U373 cells (Fig. 9A). Both the lactate production and glucose utilization in U251 and U373 cells were significantly reduced after miR-606 overexpression (Fig. 9B, C). According to the results of the CCK-8 assays, the proliferation capacity of U251 and U373 cells was significantly reduced after overexpressing miR-606 (Fig. 9D). Further, we knocked down FDX1 in U251 and U373 cells to detect the changes in the aerobic glycolysis levels and proliferative capacity of the cells. ECAR experiments showed that knockdown FDX1 significantly reduced aerobic glycolysis in U251 and U373 cells (Fig. 9E). Both lactate production and glucose utilization and decreased in U251 and U373 cells after FDX1 knockdown (Fig. 9F, G). The CCK-8 assays showed that the proliferative capacity of U251 and U373 cells was significantly reduced after FDX1 knockdown (Fig. 9H).

## Discussion

In this study, we explored the expression and functional enrichment of CRGs in glioma. Most CRGs are differentially expressed between tumor and normal tissues, and associated with glioma grade and prognosis. And we constructed the cuproptosis-related prognostic score. The AUC values of the prognostic model for predicting the 1, 3 and 5-year survival were 0.784, 0.825 and 0.786, respectively, indicating that the signature had a good performance in survival prediction. We integrated clinical information and gene features of patients from TCGA and performed the multivariable Cox regression model to develop the nomogram and calibration plots demonstrated favorable concordance between the predicted OS and the observed OS at 1, 3 and 5 years of survival.

We note that, FDX1 showed a strong positive correlation with the poor prognosis and pathological grade of glioma. FDX1 is a mitochondrial reductase involved in iron-sulfur (Fe-S) cluster formation [24, 25]. Studies have shown that FDX1 can impact the prognosis and mediate the metabolism of lung adenocarcinoma [26]. FDX1 was related with steroid metabolism and mitochondrial and may participate in the development of polycystic ovary syndrome [27]. MicroRNA (miRNA) is a small non-coding RNA of approximately 21–22 nt that regulates gene expression by identifying homologous sequences and interfering with transcription, translation, or epigenetic processes [28]. In this study, we used the miRNA microarrays datasets GSE61710 to analyze DEmiRNAs in normal brain tissue versus glioma tissues searching for targets that regulate CRGs, and found that hsa-miR-606 abnormally expressed in glioma has a binding site with FDX1 mRNA. We next found that miR-606 expression was significantly reduced in glioma tissues and cells, overexpression of miR-606 can significantly inhibit aerobic glycolysis and proliferation of GBM cells. And the mRNA and protein levels of FDX1 were significantly increased in glioma tissues and cells, knockdown of FDX1 significantly inhibit aerobic glycolysis and proliferation of GBM cells. Then we verified the binding effect and binding site of miR-606 to FDX1 mRNA by luciferase assays. However, this concept still requires more in vivo/in vitro experiments to further validate the specific regulatory mechanism. Moreover, the regulatory mechanisms and regulatory networks associated with CRGs in glioma need to be further explored.

In summary, we explored the possible prognostic value of CRGs in glioma, and explored the possible regulatory mechanism of miR-606 on FDX1, these results provide a novel insight into comprehensive glioma treatment.

## Supplementary Information

**Additional file 1: Supplementary Table 1.** Clinical characteristics of the nine samples.  **Supplementary Table 2.** Primers used for qRT-PCR. 

## Data Availability

The RNAseq data is available at in the TCGA (https://portal.gdc.cancer.gov/) GBMLGG (glioma) project. All data that support the findings of this study are available from the corresponding author upon reasonable request.

## References

[1] Xu S, Tang L, Li X, Fan F, Liu Z (2020). Immunotherapy for glioma: Current management and future application. Cancer Lett.

[2] Louis DN, Perry A, Wesseling P, Brat DJ, Cree IA, Figarella-Branger D (2021). The 2021 WHO classification of tumors of the central nervous system: a summary. Neuro Oncol.

[3] Ohgaki H, Kleihues P (2005). Population-based studies on incidence, survival rates, and genetic alterations in astrocytic and oligodendroglial gliomas. J Neuropathol Exp Neurol.

[4] Bleeker FE, Molenaar RJ, Leenstra S (2012). Recent advances in the molecular understanding of glioblastoma. J Neurooncol.

[5] DeCordova S, Shastri A, Tsolaki AG, Yasmin H, Klein L, Singh SK (2020). Molecular heterogeneity and immunosuppressive microenvironment in Glioblastoma. Front Immunol.

[6] Nicholson JG, Fine HA (2021). Diffuse glioma heterogeneity and its therapeutic implications. Cancer Discov.

[7] Tsvetkov P, Coy S, Petrova B, Dreishpoon M, Verma A, Abdusamad M (2022). Copper induces cell death by targeting lipoylated TCA cycle proteins. Science.

[8] Li SR, Bu LL, Cai L (2022). Cuproptosis: lipoylated TCA cycle proteins-mediated novel cell death pathway. Signal Transduct Target Ther.

[9] Blockhuys S, Celauro E, Hildesjö C, Feizi A, Stål O, Fierro-González JC (2017). Defining the human copper proteome and analysis of its expression variation in cancers. Metallomics.

[10] Ge EJ, Bush AI, Casini A, Cobine PA, Cross JR, DeNicola GM (2022). Connecting copper and cancer: from transition metal signalling to metalloplasia. Nat Rev Cancer.

[11] Ishida S, Andreux P, Poitry-Yamate C, Auwerx J, Hanahan D (2013). Bioavailable copper modulates oxidative phosphorylation and growth of tumors. Proc Natl Acad Sci U S A.

[12] Babak MV, Ahn D (2021). Modulation of intracellular copper levels as the mechanism of action of anticancer copper complexes: clinical relevance. Biomedicines.

[13] Feng Y, Zeng JW, Ma Q, Zhang S, Tang J, Feng JF (2020). Serum copper and zinc levels in breast cancer: a meta-analysis. J Trace Elem Med Biol.

[14] Wang W, Wang X, Luo J, Chen X, Ma K, He H (2021). Serum copper level and the copper-to-zinc ratio could be useful in the prediction of lung cancer and its prognosis: a case-control study in Northeast China. Nutr Cancer.

[15] Khoshdel Z, Naghibalhossaini F, Abdollahi K, Shojaei S, Moradi M, Malekzadeh M (2016). Serum copper and zinc levels among Iranian colorectal cancer patients. Biol Trace Elem Res.

[16] Vivian J, Rao AA, Nothaft FA, Ketchum C, Armstrong J, Novak A (2017). Toil enables reproducible, open source, big biomedical data analyses. Nat Biotechnol.

[17] Hänzelmann S, Castelo R, Guinney J (2013). GSVA: gene set variation analysis for microarray and RNA-seq data. BMC Bioinformatics.

[18] Bindea G, Mlecnik B, Tosolini M, Kirilovsky A, Waldner M, Obenauf AC (2013). Spatiotemporal dynamics of intratumoral immune cells reveal the immune landscape in human cancer. Immunity.

[19] Ceccarelli M, Barthel FP, Malta TM, Sabedot TS, Salama SR, Murray BA (2016). Molecular profiling reveals biologically discrete subsets and pathways of progression in diffuse glioma. Cell.

[20] Liu J, Lichtenberg T, Hoadley KA, Poisson LM, Lazar AJ, Cherniack AD (2018). An Integrated TCGA pan-cancer clinical data resource to drive high-quality survival outcome analytics. Cell.

[21] Kanehisa M, Goto S (2000). KEGG: kyoto encyclopedia of genes and genomes. Nucleic Acids Res.

[22] Kanehisa M (2019). Toward understanding the origin and evolution of cellular organisms. Protein Sci.

[23] Kanehisa M, Furumichi M, Sato Y, Kawashima M, Ishiguro-Watanabe M. KEGG for taxonomy-based analysis of pathways and genomes. Nucleic Acids Res. 2023;51(D1):D587–92. 10.1093/nar/gkac963 PMC9825424 36300620

[24] Cai K, Tonelli M, Frederick RO, Markley JL (2017). Human mitochondrial Ferredoxin 1 (FDX1) and Ferredoxin 2 (FDX2) both bind cysteine desulfurase and donate electrons for iron-sulfur cluster biosynthesis. Biochemistry.

[25] Sheftel AD, Stehling O, Pierik AJ, Elsässer HP, Mühlenhoff U, Webert H (2010). Humans possess two mitochondrial ferredoxins, Fdx1 and Fdx2, with distinct roles in steroidogenesis, heme, and Fe/S cluster biosynthesis. Proc Natl Acad Sci U S A.

[26] Zhang Z, Ma Y, Guo X, Du Y, Zhu Q, Wang X (2021). FDX1 can impact the prognosis and mediate the metabolism of lung adenocarcinoma. Front Pharmacol.

[27] Wang Z, Dong H, Yang L, Yi P, Wang Q, Huang D (2021). The role of FDX1 in granulosa cell of Polycystic ovary syndrome (PCOS). BMC Endocr Disord.

[28] Chen L, Heikkinen L, Wang C, Yang Y, Sun H, Wong G (2019). Trends in the development of miRNA bioinformatics tools. Brief Bioinform.

