# Supplementary material for: A novel cuproptosis-related gene signature to predict prognosis in Glioma
Source: BMC Cancer. 2023 Mar 13;23:237. doi: 10.1186/s12885-023-10714-8 (PMC10012466; doi:10.1186/s12885-023-10714-8)

Supplementary Table 1. Clinical characteristics of the nine samples

	Sample	Age	Sex	Grading	Pathological features	Molecular/genetic signatures
NBT	Sample 1	80	male	n/a	n/a	n/a
	Sample 2	73	female	n/a	n/a	n/a
	Sample 3	58	male	n/a	n/a	n/a
LGG	Sample 4	27	male	WHO grade II	The tumor cells were round, denser, with no obvious necrosis, and insignificant vascular proliferation.	GFAP (+); Olig-2 (+); CD34 (vascular +); Syn (+); IDH-1 (+); P53 (~30% +); Vimentin (residual neurons +); NeuN (-); H3K27M (-); H3K27me3 (+); Ki-67 (~5% +).
	Sample 5	47	male	WHO gradeII	Tumor cells were seen as small round shapes with diffuse sheet-like dense arrangements and scattered small foci of calcification.	GFAP(+); Olig-2(+); Vimentin(-); Syn(+); NeuN(-); EMA(small amount of paranuclear dotted+); IDH-1(-); H3K27me3(-); P53(approximately 5%+, no abnormal expression); Ki-67(approximately 5%+); H3K27M(-); CD34(+).
	Sample 6	55	female	WHO grade II	Glial cells were proliferating, densely arranged, and with a translucent cytoplasm.	H3K27me3(+); Olig-2(+); Vimentin(-); CD56(+); CK(-); CD34(-); EMA(-); H3K27M(-); GFAP(-); IDH-1(+); Ki-67(10%+); P53(5%+); NeuN(-); Syn(+).
HGG	Sample 7	50	female	WHO grade IV	The tumor cells were densely arranged, with marked vascular proliferation and some tissue necrosis and calcification.	EMA (+); Vimentin (partial +); GFAP (+); Olig-2 (partial +); IDH-1 (-); S-100 (focal +); Ki-67 (approximately 15% +); CD34 (vascular +); BRAF (-); Inhibin (-); NSE (focal +); NeuN (-); H3K27M(-); H3K27me3(+).
	Sample 8	67	male	WHO grade IV	Cells were shuttle-shaped or short shuttle-shaped, arranged in sheets, with the proliferation of small vessels, and presenting as hemorrhagic necrosis.	GFAP (+); Vimentin (+); PR (-); EMA (focal weak +); Syn (weak +); CD34 (vascular +); S-100 (partial +); Olig-2 (+); NeuN (-); Stat6 (-); IDH-1 (focal +); H3K27M (-); H3K27me3 (+); P53 (~30%+); Ki-67 (focal ~10%+).
	Sample 9	56	female	WHO grade IV	Tumor cells were of variable sizes and arranged in sheets; tumor giant cells were observed, and necrosis was visible.	GFAP (+); Vimentin (partial +); CD34 (+); EMA (partial +); Olig-2 (partial +); Syn (partial +); NeuN (focal +); H3K27M (-); H3K27me3 (focal +); P53 (approximately 80% +); Ki-67 (30–40% +); IDH-1 (-).

Supplementary Table 2. Primers used for qRT-PCR

Gene	Sequence (5'->3') or Assay ID
miR-606	F: AGCCAGCGAAACTACTGAAAATCAA
FDX1	F: TTCAACCTGTCACCTCATCTTTG
	R: TGCCAGATCGAGCATGTCATT
β-actin	F: ATTCCTATGTGGGCGACGAG
	R: GCCACACGCAGCTCATTGTA

Full length uncropped original western blots used in the manuscript

Figure 8E

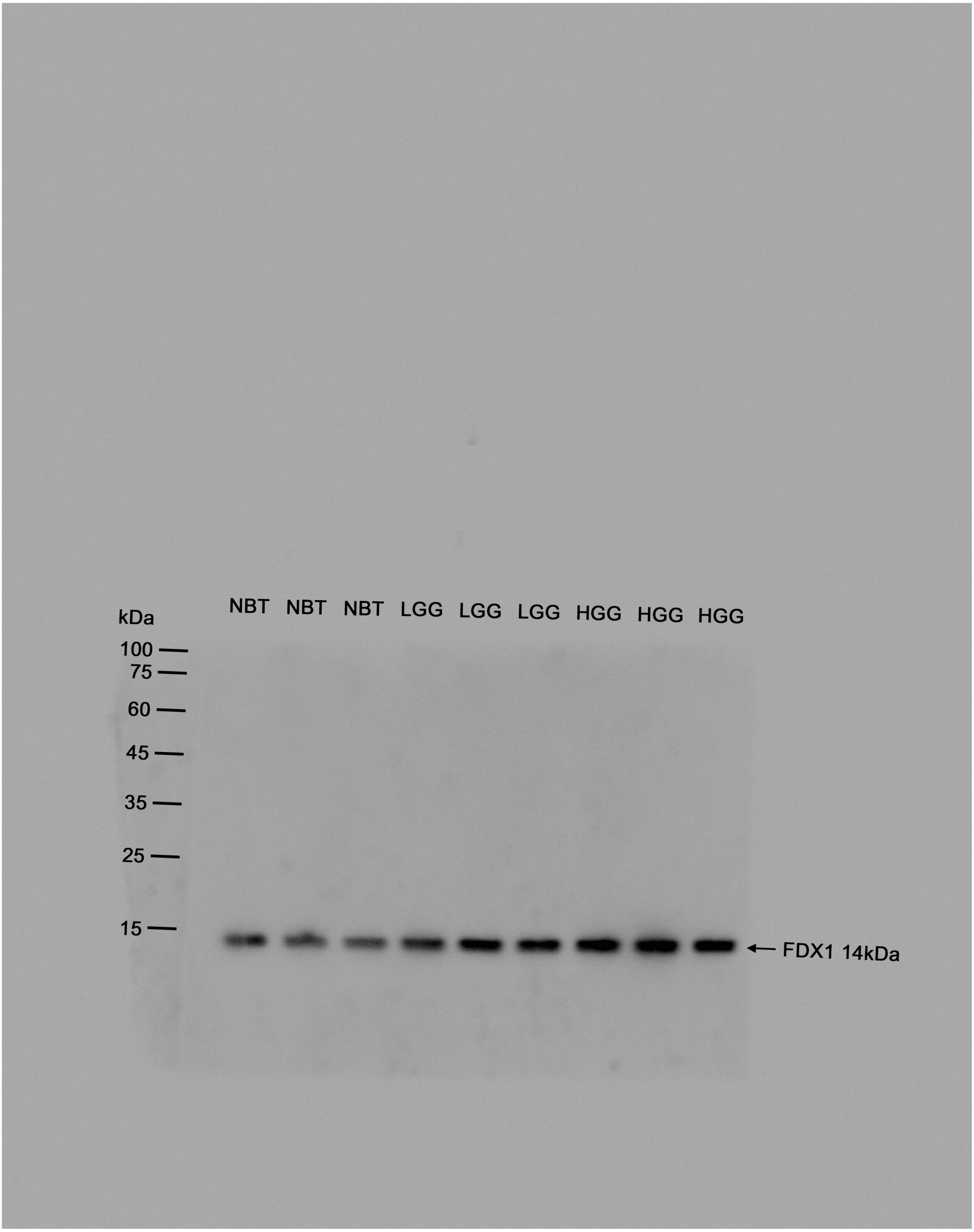

Figure 8E

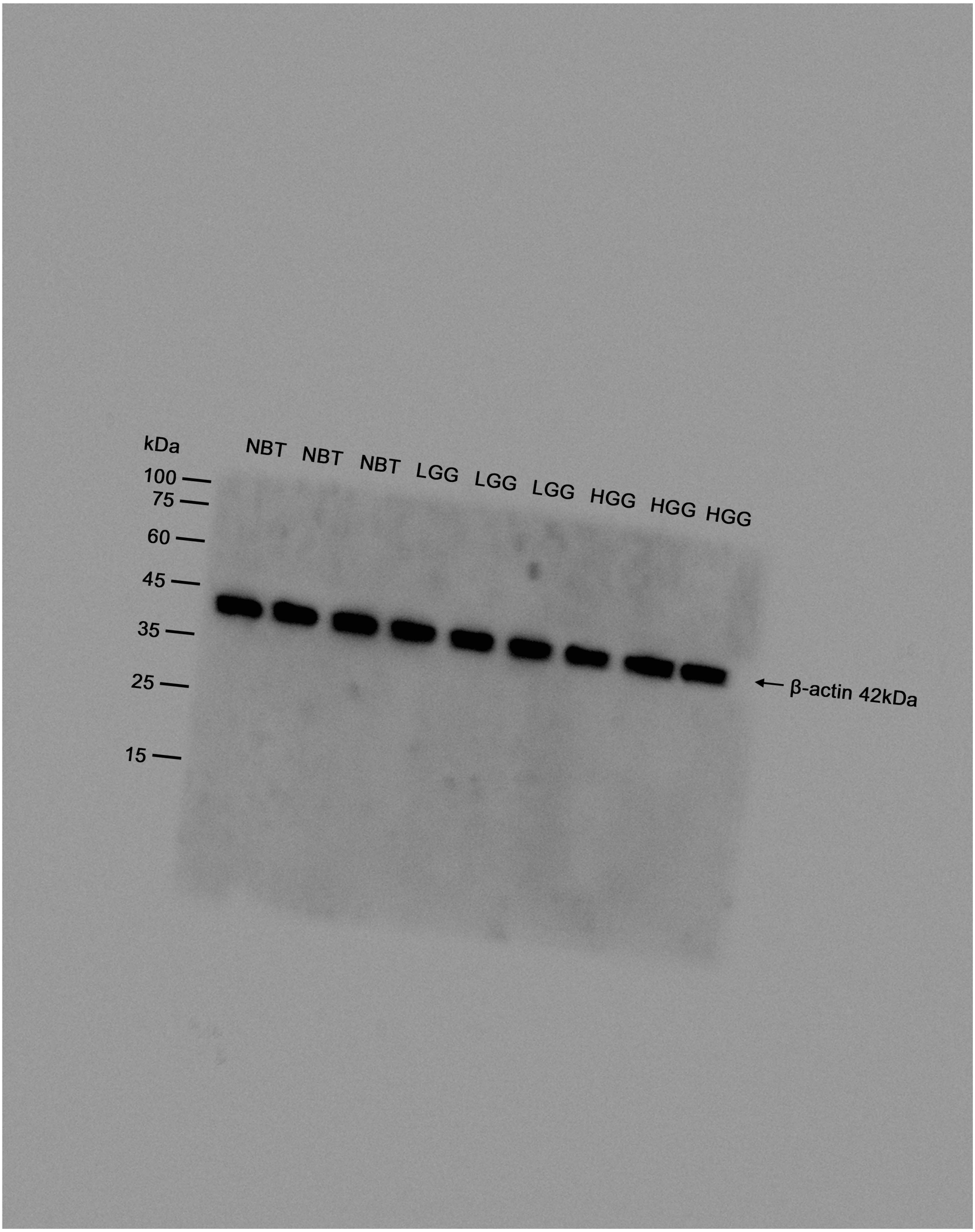

kDa
NBT
NBT
NBT
LGG
LGG
LGG
HGG
HGG
HGG
100
75
60
45
35
25
15
β-actin 42kDa

Figure 8F

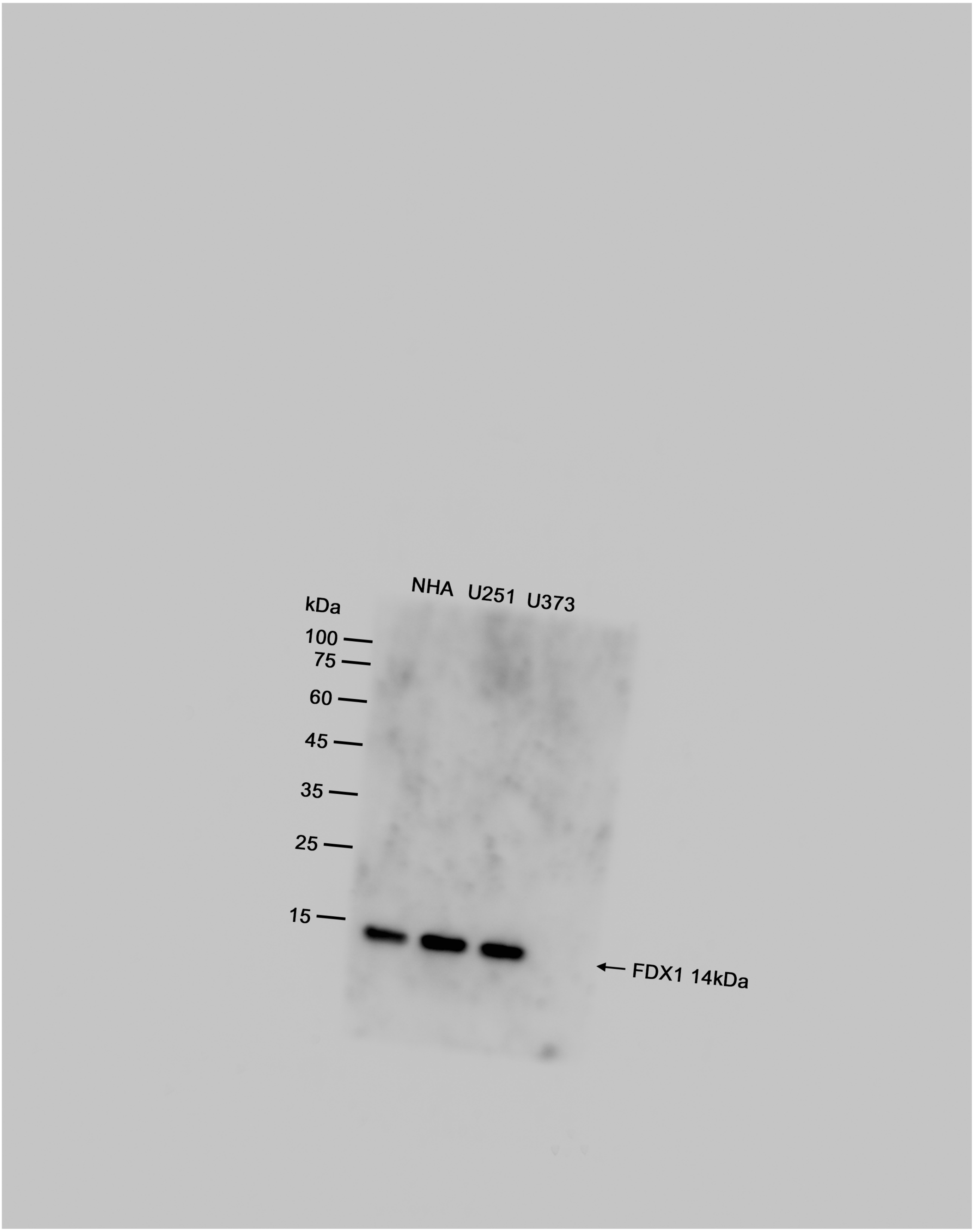

Figure 8F

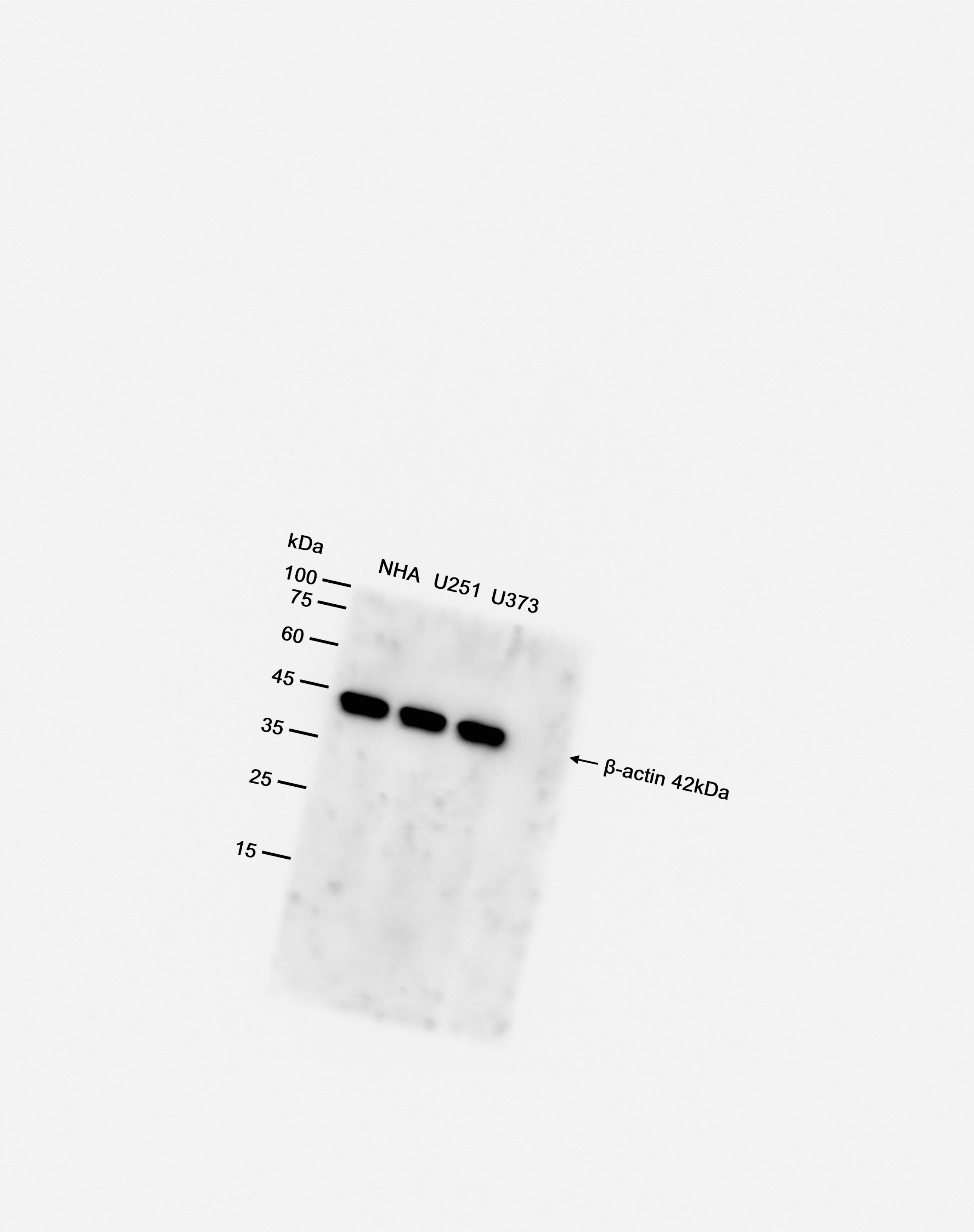

Supplement: Supplementary file 1 — Additional file 1: Supplementary Table 1. Clinical characteristics of the nine samples. Supplementary Table 2. Primers used for qRT-PCR. [file 12885_2023_10714_MOESM1_ESM.pdf]
